# Molecular Mechanisms of Action of Novel Psychoactive Substances (NPS). A New Threat for Young Drug Users with Forensic-Toxicological Implications

**DOI:** 10.3390/life11050440

**Published:** 2021-05-14

**Authors:** Arianna Giorgetti, Jennifer P. Pascali, Paolo Fais, Guido Pelletti, Andrea Gabbin, Giorgia Franchetti, Giovanni Cecchetto, Guido Viel

**Affiliations:** 1 Unit of Legal Medicine, Department of Medical and Surgical Sciences, University of Bologna, Via Irnerio 49, 40126 Bologna, Italy; arianna.giorgetti@unibo.it (A.G.); paolo.fais@unibo.it (P.F.); guido.pelletti2@unibo.it (G.P.); 2 Department of Cardiac, Thoracic, Vascular Sciences and Public Health, University of Padova, Via Falloppio 50, 35121 Padova, Italy; jennifer.pascali@unipd.it (J.P.P.); andrea.gabbin@studenti.unipd.it (A.G.); giorgia.franchetti@studenti.unipd.it (G.F.); giovanni.cecchetto.1@unipd.it (G.C.)

**Keywords:** forensic toxicology, new psychoactive substances (NPS), mass spectrometry, toxicodynamic, mechanism of action

## Abstract

Novel psychoactive substances (NPS) represent a severe health risk for drug users. Even though the phenomenon has been growing since the early 2000s, the mechanisms of action of NPS at the receptors and beyond them are still scarcely understood. The aim of the present study was to provide a systematic review of the updated knowledge regarding the molecular mechanisms underlying the toxicity of synthetic opioids, cannabinoids, cathinones, and stimulants. The study was conducted on the PubMed database. Study eligibility criteria included relevance to the topic, English language, and time of publication (2010–2020). A combined Mesh and free-text protocols search was performed. Study selection was performed on the title/abstract and, in doubtful cases, on the full texts of papers. Of the 580 records identified through PubMed searching and reference checking, 307 were excluded by title/abstract and 78 additional papers were excluded after full-text reading, leaving a total of 155 included papers. Molecular mechanisms of synthetic opioids, synthetic cannabinoids, stimulants, psychedelics, and hallucinogens were reviewed and mostly involved both a receptor-mediated and non-receptor mediated cellular modulation with multiple neurotransmitters interactions. The molecular mechanisms underlying the action of NPS are more complex than expected, with a wide range of overlap among activated receptors and neurotransmitter systems. The peculiar action profile of single compounds does not necessarily reflect that of the structural class to which they belong, accounting for possible unexpected toxic reactions.

## 1. Introduction

Novel Psychoactive Substances (NPS) are an inhomogeneous group of substances which are typically sold as “legal” alternatives to the classical scheduled drugs of abuse, such as heroin, cocaine, amphetamines, benzodiazepines etc. [1]. The term “novel” derives from the fact that, contrarily to classical drugs of abuse, NPS were not covered by the International Drug Control Conventions of 1961–1971 [1,2]. Nowadays, the term could be considered somehow misleading, since many of the compounds have been later included in the list of scheduled substances at a national or international level [2]. Nonetheless, the “legality” of these compounds still represents one of the main attractions for consumers [2]. One of the characteristics of the NPS phenomenon resides in the ease of producing novel compounds by minimal twisting or modifications of the chemical structures, producing a nonscheduled molecule and circumventing existing legislations. Some authors have underlined that the huge efforts of national/international organizations, striving to include a molecule within the list of prohibited substances, are the main trigger for the innovation and production of novel compounds (the so-called “cat and mouse model”) [2,3], which have rated more than 50 novel compounds per year since early 2000. Thus, even if many of these substances are now controlled, several are still nonscheduled, undetected, and unidentified. These substances are not even consumed or produced, but certainly will be in the next future. To date, the European Monitoring Centre for Drugs and Drug Addiction (EMCDDA) has monitored 790 new psychoactive substances [1,4,5]. The main drives for consuming NPS are also the reported “safety” and “natural origin” by the supplier, both concepts that have led to an extraordinary growth in popularity of NPS since 2007, especially among younger users browsing the Internet [5,6,7]. Although they are claimed as “safe” or sold “not for human consumption,” these substances pose severe health risks, the prevention of which cannot disregard from an in-depth understanding of their pharmacokinetic and pharmacodynamic properties.

The aim of the present review is to provide an overview of the molecular mechanisms of action of the main classes of novel psychoactive substances (synthetic opioids, synthetic cannabinoids, and synthetic cathinones and stimulants) to better understand the health risks and the effects arising from their consumption, according to the PICOS process:

P—population/problem: Novel psychoactive substances, synthetic opioids, synthetic cannabinoids, synthetic cathinones, and stimulants;

I—intervention: In vivo or in vitro studies;

C—comparison, control: Previous knowledge;

O—outcome of interest: Description of the molecular mechanisms of action of novel psychoactive substances;

S—study design: PubMed review.

## 2. Materials and Methods

A recent literature search was conducted on PubMed-indexed articles through both Mesh terms and free-text protocols, pairing any included NPS term with any included “mechanism of action,” according to the PRISMA Flow diagram.

### 2.1. Search Term

*Novel Psychoactive Substances*. The following terms were searched: Novel psychoactive substance(s); new psychoactive substance(s); synthetic opioids; synthetic cannabinoids; synthetic cathinones; (“Synthetic Drugs” [Mesh]) AND (“Cannabinoids” [Mesh] OR “Analgesics, Opioid” [Mesh]).

*Molecular mechanisms*. The terms “mechanism,” “mechanism of action,” and “receptor” were alternatively used.

### 2.2. Eligibility Criteria

The English language and time interval of publication, from January 2010 to December 2020, were applied as filters and inclusion criteria. Eligible studies investigated the receptor-based mechanism of action of NPS, and particularly of synthetic cathinones and stimulants, synthetic cannabinoids, and synthetic opioids, or the neural mechanism through which these substances exert their effects on the brain. In vivo and ex vivo studies were also included.

### 2.3. Exclusion Criteria

Papers focusing on the potential therapeutic effects of NPS were not considered in the present review.

### 2.4. Study Selection and Data Collection Process

Titles and abstracts were first screened for inclusion criteria and, in dubious cases, full texts were examined. References of the selected article were further screened, and related papers were included as a source of additional data.

A database was built with the included articles. Authors, title, journal, and year of publication were extracted, and papers were considered for the respective population and outcomes of interest.

## 3. Results

**Figure 1:**
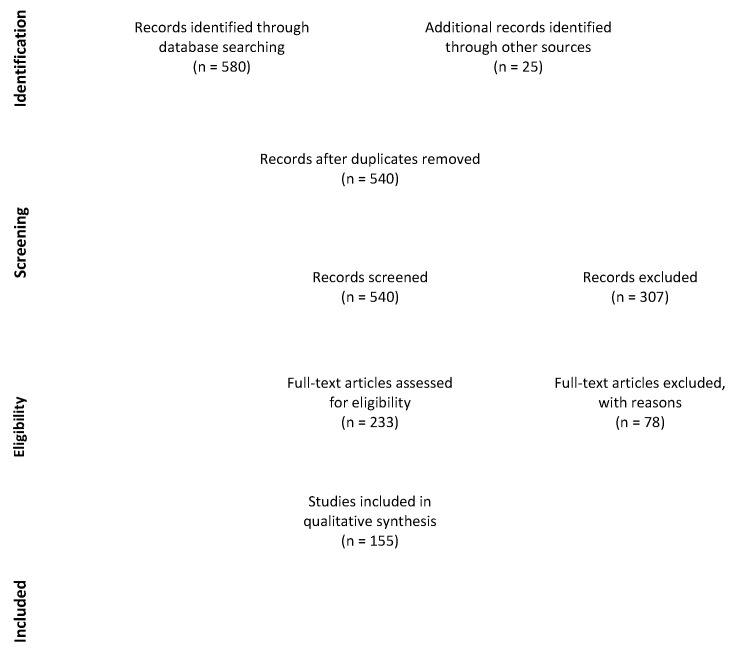
PRISMA flow diagram of the present review. From: Moher D, Liberati A, Tetzlaff J, et al. Preferred reporting items for systematic reviews and meta-analyses: the PRISMA statement. PLoS medicine, 2009, 6(7): e1000097 [8].

The literature search provided the following results (Figure 1).

Overall, 155 studies were included, of which 22 (22/155, 14.2%) used new or novel psychoactive substances terms. Among single NPS classes, synthetic cannabinoids yielded the majority of papers, with 77 included articles (77/155, 49.7%). In total, 17 articles were included for synthetic opioids (17/155, 11.0%) and 14 (14/155, 9.0%) for synthetic cathinones. In addition, 25 papers (25/155, 16.1%) were included from references.

Overall, 155 papers were included in the present review, and discussed by “population” and “outcome” of interest.

### 3.1. Synthetic Opioids

Although they are still of limited diffusion across the European market, synthetic opioids represent a massive health risk due to their high potency and severe adverse effects. Indeed, they have been reported as one of the main causes of the waves of opioid deaths in the USA [9,10,11,12,13]. The term “synthetic opioids” includes a wide range of antinociceptive and analgesic compounds (fentanyl derivatives, benzamide, acetamide and piperazine families) [14] that act as partial or full agonists at G-protein-coupled receptors (μ, κ, and δ) [15,16,17]. μ-opioid receptors, as shown in knock-out mice, are mainly located in brain and gastrointestinal tract and lead to anxiolysis, relaxation, sedation, antinociception, euphoria, and respiratory depression [7,17,18,19,20,21]. Other effects include hypothermia, miosis, nausea, and the inhibition of gastrointestinal propulsion. The activation of κ and δ-receptors also leads to hallucination, dissociate feelings, and dysphoria, as shown for U-50488H, and immunomodulation [14,22,23]. The peculiar profile of opioid receptor agonism might explain also unusual toxicity, e.g., a deep level of unconsciousness for MT-45 [21]. G-proteins (Gα_i_), determining the inhibition of cyclic adenosine monophosphate (cAMP) production, inhibition of Ca^2+^ channels of the L-type, and activation of the inward-rectifying K^+^ channels, leading to hyperpolarization and reduced neuronal excitability, are mainly responsible for analgesia, while β-arrestins are additional transducers, which could be involved in the unwanted effects of synthetic opioids [24].

Generally, synthetic opioids present stronger analgesic activity compared to morphine and classical opioid. Fentanyl and carfentanyl are approximately 50–100- and 10000-times respectively more potent than classical opioids [25,26,27,28]. Affinity to opioid receptors significantly differs between stereoisomers, e.g., only the trans form has opioid activity for U-47700 and U-50488 [27], and R-enantiomers are thought to be more potent than the S ones [29]. Even though the in vitro efficacy and potency of several new compounds, such as AP-237, bromadol, brorphine, tianeptine, isotonitazene, and piperidylthiambuetene, has been characterized [9,30], their exact psychopharmacological and neurotoxicological profiles remain scarcely known [25].

Synthetic opioids might interact also with other receptors, especially with the serotoninergic ones or with monoamine transporters such as norepinephrine transporter (NET) and serotonin transporter (SERT) [7], as seen for AH-7921, the effects of which were prolonged by the co-injection of serotonin (5HT) and attenuated by norepinephrine [31]. Contrarily to morphine, which has antagonistic interactions with 5HT_3A_ receptors [32], interaction of fentanyl with 5HT_1A_ and _2A_ receptors might lead to additional toxicity due to serotonin syndrome, especially in combination with other drugs active on the serotonin system [33]. This might explain why rescue therapy with naloxone (receptor antagonist) are noneffective, or less effective than what expected [34,35,36].

Fentanyl and carfentanil also showed relevant affinity for α1 adrenoceptors, possibly explaining severe muscle rigidity at the laryngeal, tracheal, and chest musculature and the closure of vocal cords, as well as for dopamine receptors (D4.4 and D1). Moreover, they blocked the uptake by monoamine transporter 2 and this might further explain the relevant respiratory and cardiothoracic effects [37].

### 3.2. Synthetic Cannabinoids

**Figure 2:**
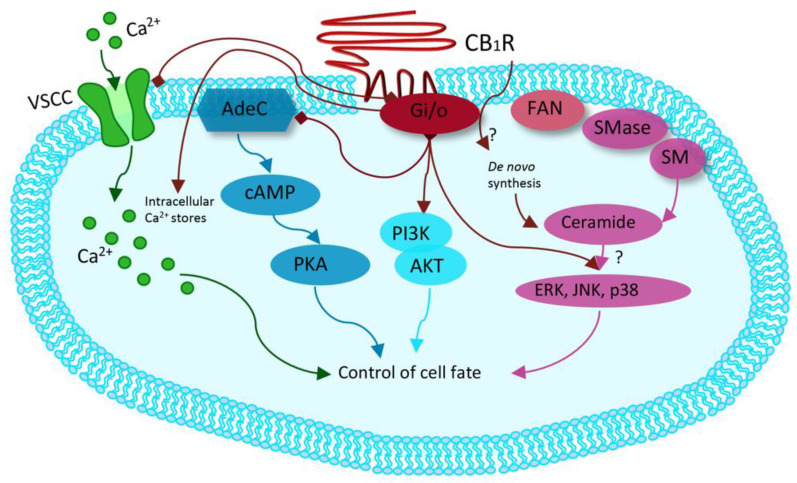
Modified from Guzman et al. Cannabinoids: Potential anticancer agents. *Nat Rev Cancer*. Mechanism activated by the receptor of human cannabinoids 1 (CB1R), ranging from binding to G-protein-coupled receptors (Gi/o) with inhibition of the adenylyl cyclase (AdeC), and therefore of the cyclicAMP (cAMP) and of the protein kinase A (PKA). Inhibition of voltage-sensitive Ca^2+^ channels (VSCC); release of Ca^2+^ from intracellular stores; activation of the phosphatidylinositol 3-kinase (PI3K)–AKT pathway; activation of mitogen-activated protein kinase cascades as extracellular-signal-regulated kinase (ERK), JUN amino-terminal kinase (JNK), and p38 and ceramide generation through FAN–sphingomyelinase (factor associated with neutral sphingomyelinase activation–SMase).

Synthetic cannabinoids, also called “Spice,” are synthetic cannabinoid receptor agonists (SCRAs) which have been originally developed for their potential therapeutic role by exploiting the endocannabinoid system [38,39,40]. Since then, “Spice” products have been sold as legal marijuana surrogate, becoming very popular among younger people and now representing the widest class of NPS. Synthetic cannabinoids are full agonists at CB_1_ and CB_2_, G-coupled human cannabinoid receptors [41,42,43,44,45,46,47,48,49], which are weakly bound by delta-9-tetrahydrocannabinol (THC) and which inhibit adenylyl cyclase and activate mitogen-activated protein kinases [50,51]. CB receptors can also activate inwardly, rectifying potassium channels and mediating an inhibition of N- and P/Q-type calcium currents (more details are given in Figure 2 [50].

CB_1_ receptors are mainly located in the central nervous system, thus covering most of the psychoactive effects of SCRAs. Due to the distribution of CB_1_ and CB_2_ receptors on the terminals of neuron, which mediate a modulation and inhibition of synaptic transmission, cannabinoids have effects on neuronal development, motor function, cognition, and memory, appetite, sleep, thermoregulation, analgesia, reward processes, cardiovascular, respiratory, immune, and reproductive functions [7,52,53,54,55]. Reward, euphoria, memory loss, altered vigilance, anxiety and cognitive deficit, proconvulsant, antinociceptive, cataleptic, hypolocomotion, and hypothermic effects of SCRAs, such as JWH-018, JWH-073, 5F-AMB, 5F-AB-PINACA, and Cumyl-4CN-BINACA, are mediated by CB_1_ receptor activation, as demonstrated in CB_1_ knock-out mice or by CB_1_-blocking agents [56,57,58,59,60,61,62,63,64,65]. These neurological effects differ from that of classical cannabinoids, e.g., cannabidiol (CBD), one of the main non-psychotropic cannabinoids, which has been shown to interact with peroxisome proliferator-activated receptors and acetylcholinesterase and to modulate beta-amyloid deposition and tau protein phosphorylation, with several promising therapeutic uses [66].

In adolescent and adult mice, in vivo brain administration of 5-MDMB-PICA produced anxiety-like and compulsive states [67]. The effects on neuronal development have been also studied. Brain malformations have also been shown due to inhibition of Pax-6, which is necessary for the closure of the nascent neural tube, as well as CB_1_-mediated ocular malformation, lack of memory retention and hyperactivity, and inhibition of new synapses formation in hippocampal neurons [68]. Moreover, SCRAs induced hyperreflexia and myoclonias, not induced by THC, with effects prevented by the administration of CB_1_ receptor antagonist/reverse agonist AM 251, while this is not the case for sensory-motor impairments [69,70]. CB receptor antagonists also prevent SCRAs from producing cytotoxic effects on cytotrophoblasts cells, forebrain cultures, and skeletal muscle cells by CP-55.940 and CP 47.497-C8 [71,72,73,74,75].

CB_1_ receptor have been shown to have a role in the interaction between ethanol and SCRAs, with an increase in ethanol-induced motor impairments after JWH-018 administration [76], and in analgesia, with a synergistic effect between SCRAs and opioids [77].

The selectivity, affinity, and activity of SCRAs appear to be related to their chemical structure [60,78,79,80], e.g., the fluorination of the alkyl side chain of Cumyl-PEGACLONE led to a more affine and active compound, 5F-Cumyl-PEGACLONE [81]. The pharmacological profile (affinity and activity) of 5F-Cumyl-PICA 5F-Cumyl-PINACA and 5F-Cumyl-P7AICA has been also recently determined [82]. Halogenated JWH-018 was less effective in causing seizures, myoclonia, and hyperreflexia than JWH-018 [83]. Moreover, the enantiomeric configuration might have a role in the affinity to receptors [84,85].

One of the main issues of SCRAs, which might also lead to death, is represented by cardiotoxicity and cannabinoid-receptor associated arrhythmias [86], which might be a CB_2_-mediated effect, resulting in prolonged QT interval [87]. CB_2_ might also mediate a vasodilator effect, additionally triggered by independent (nitric-oxide-related) mechanisms [88]. However, no chronotropic effect by CB_2_ was shown on isolated rat atria treated with SCRAs, and the exact mechanism of SCRAs-related arrhythmias remains unknown [89,90].

Metabolites have been shown to retain activity at CB_1_ and/or CB_2_ receptors [43] as shown for JWH-018, JWH-073, 5F-AKB48, and AB-PINACA, with implications for toxicity [91,92,93,94]. However, a non-receptor-mediated mechanism has been proposed for the toxicity of the JWH-018 main metabolite when compared to the parent drug, and for WIN55,212-2 in spatial memory tasks, which causes a CB-receptor-independent decrease of cholinergic activation [95,96].

Interactions with other neuroceptors, leading to inhibition of cholinergic contraction in the respiratory system, inhibition of glutamate release, and release of dopamine in the nucleus accumbens, leading in vivo to abuse potential and psychomotor agitation, might be partly explained by a presynaptic CB_1_ mediated effect [97,98,99]. Interactions of SCRAs has been described with dopamine, serotonin, and glutamate systems, with possible effects on schizophrenia and psychosis after SCRAs intake [100]. Other non-cannabinoid-mediated interactions include those with other G-coupled protein receptors, capsaicin receptor, and the vanilloid receptor 1 [52,101,102]. It should be mentioned that transient receptor potential (TRP) channels might also mediate significant effects of SCRAs, since endogenous endocannabinoids such as anandamide are TRP agonists [103]. Moreover, as shown for AM2201 and JWH-018, SCRAs might act as allosteric modulators of other receptors, e.g., 5-HT_1A_ receptors, determining a hypothermic response in mice lacking CB receptors [104] or producing behavioral responses [105]. SCRAs such as WIN55,212-2 can also inhibit a 5-HT mediated current in a non-CB-receptor-dependent manner [106].

### 3.3. Stimulants, Psychedelics, and Hallucinogens

Stimulants such as cocaine, amphetamine, MDMA, and cathinones typically determine a sympathomimetic action, with tachycardia and hypertension, hallucinogenic (including psychosis and delirium), and psychoactive stimulants effects, e.g., agitation, euphoria, and increased emotional empathy [7,107,108,109,110,111,112]. Novel stimulants are considered to lead to the same effects, though with higher potency [113,114], by interacting with monoamine transporters, particularly with dopamine transporter (DAT), NET, and SERT. This interaction might be of the “blocking type,” i.e., by inhibition of the uptake of neurotransmitter from the extracellular space, thus leading to an increase of the respective monoamines [115]. In addition or alternatively to the blocking of monoamine transporters, some drugs might act as “substrates,” entering the intracellular space, releasing monoamine, and mediating a so-called non-exocytotic monoamine efflux, as occurs for MDMA and methamphetamine [7,115,116,117,118].

**Figure 3:**
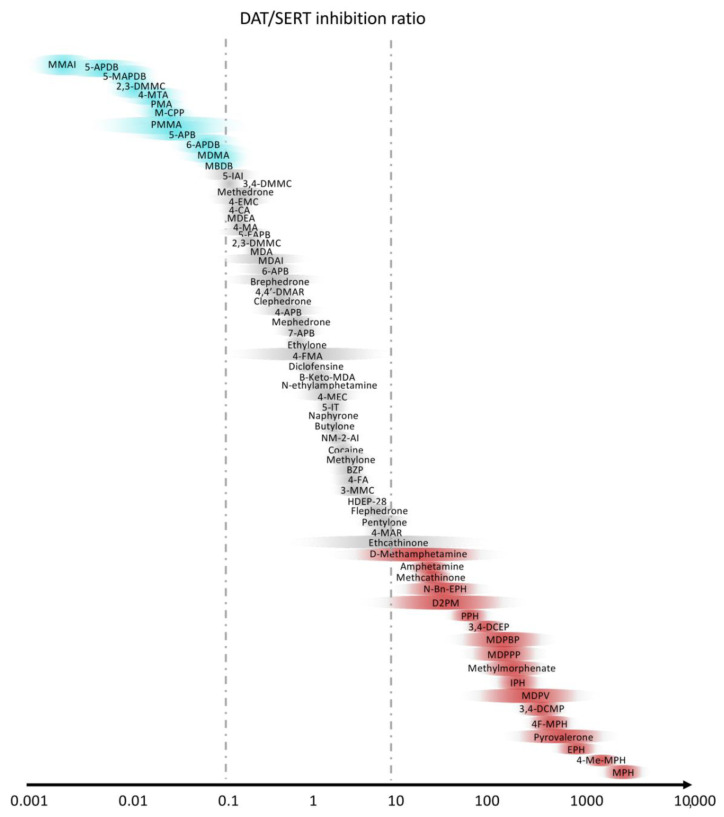
Selectivity of stimulants with the ratio between dopamine (DAT) and serotonin (SERT) transporters. Slightly modified from Luethi et al. [7].

Novel psychostimulant drugs are mostly classified on the basis of the greater noradrenergic vs. dopaminergic vs. serotoninergic activity [119,120,121,122,123]. Indeed, a high DAT/SERT ratio and a substrate-type monoamine releasers action is typical of amphetamine-type stimulant-like properties, with high potential of abuse [124], whereas a lower ratio (0.01–0.1) leads mainly to empathogenic effects, similarly to MDMA, with low intracranial self-stimulation [7,116,125]. The DAT/SERT ratios of the main stimulants are shown in Figure 3.

Serotonergic compounds usually lead to a subjective sense of well-being and increased sociability in humans. These compounds have been associated with 5-HT syndrome, hyperthermia, and resulting organ failure [116]. Hyperthermia might be reduced using adrenergic antagonists, highlighting the importance of adrenergic receptors in the determination of this adverse effect [126].

The effects of psychostimulants seem to be also influenced by the chiral configuration, e.g., S-enantiomer may have greater serotoninergic features, and R-enantiomers may have higher dopaminergic features [127].

Amphetamines are substrates of vesicular monoamine transporters and inhibitors of monoamine oxidases and interact with trace amine-associated receptor 1 (TAAR1) [7,112,128,129,130,131]. Stimulants also present complex interactions with neuroendocrine molecules, e.g., they increase oxytocin levels, although the latter, as demonstrated for 4-Fluoroamphetamine, might be unrelated to cognitive and emotional behavior and empathy [132]. Amphetamine-type psychostimulant include derivatives of aminorex, such as 4-methylaminorex (4-MAR) and 4,4′-dimethylaminorex (4,4′-DMAR) [133,134]. Although both are derivatives of aminorex, the former appears as a more typical stimulant, with a high DAT/SERT ratio, while the latter is thought to lead mainly to empathogenic effects [125].

Both 3,4-dichloromethylphenidate (3,4-CTMP) and ethylphenidate are analogs of methylphenidate, a prescription drug used in the treatment of the attention-deficit hyperactivity disorder (ADHD), and are commonly consumed to produce euphoria or as cognitive enhancers [7,135]. Even though 3,4-CTMP was originally studied as a treatment for cocaine abuse [136], methylphenidate derivatives determine a dopamine and a noradrenaline efflux in the nucleus accumbens and stria terminalis, which are involved in the hedonic processing system and which explain the abuse potential of the drugs, with NET and DAT inhibitor activity [7]. 3,4-CTMP is mainly considered as a a “cocaine-like” instead of “amphetamine-like” drug, since it increases the release of dopamine when stimulated, but not in baseline conditions [135]. As a transporter inhibitor, diclofensine has also a similar pharmacological profile to cocaine. However, it also has high affinity for D_2_ and for adrenergic α_1A_ and α_2A_ receptors [137].

Phenmetrazines derivatives, e.g., 3-fluorophenmetrazine (3-FPM), diphenylprolinol (D2PM), and desoxypipradrol (2-DPMP), similarly to methylphenidate, are DAT and NET inhibitors, with prolonged psychostimulants effects and low serotoninergic effects [7,116].

Synthetic cathinones, typically called “bath salts,” are both indirect releasers by transporter blocking action, e.g., pyrovalerone derivatives, and direct substrate effects, e.g., 4-methylmethcathinone (mephedrone) and methylone [7,118,138,139,140]. Pyrrolidine-containing cathinones, such as methylenedioxypyrovalerone (MDPV,) are blockers at DAT and NET with lower potency at SERT and do not show a substrate activity [118]. MDPV, one of the most popular bath salts, has been shown to induce an EEG synchronization associated with delirium syndrome in rats treated by microdialysis, blocked by D_1_ and D_2_ receptor antagonists [141]. Moreover, it led to the reduction of social play behavior in young adult male rats, while effects were blocked by RX821002 and flupenthixol, respectively, α_2_ and dopamine receptor antagonists [142]. Drug-induced dopaminergic activity parallels the locomotor stimulation and rewarding effect [118,143,144]. Methylone is a nonspecific substrate [118], producing an inward current at SERT but not at DAT, similarly to MDMA [145], and oxidative stress, which is responsible for the neurotoxicity of methylone and, to a greater extent, MDPV [146]. 4-MEC, 4-MePP, and α-PVP also mainly block DAT, with greater abuse potential compared to other stimulants [147,148]. Unusual neuropsychiatric symptoms have been attributed to some synthetic cathinones, suggesting additional pharmacological features. Among synthetic cathinones, α-pyrrolidinohexiophenone (α-PHP) also exhibit anticholinergic activity (at M_1_ and M_2_ receptors), which might have a role in clinical features such as delusions, cognitive impairment, and cardiovascular effect such as tachycardia and hypertension [149]. α-PPP has an antagonistic interaction with 5-HT_2A_-receptors, which could be responsible for its limited abuse potential compared to other compounds of the same class [150].

Among benzofurans (e.g., 5-APB) indole derivatives and amino-indane, 5-iodoaminoindane (5-IAI), and 5,6-methylenedioxy-2-aminoindane (MDAI) preferentially inhibit SERT and NET, and the latter also has shown NE-releasing properties [116,151,152,153,154,155]. Among piperazines, 1-benzylpiperazine (BZP) has a more selective action on NET, with no or low serotoninergic effects, leading to cardiostimulant effects, agitation, seizures, and hyperthermia, while other compounds pertaining to the same class, e.g., meta-chlorophenylpiperazine (m-CPP) and trifluoromethylphenylpiperazine (TFMPP), have low effects on DAT and NET and predominantly act as indirect (transporter inhibitor) and direct serotonergic agonists, resulting in effects such as dysphoria, dizziness, anxiety, and more nausea compared to MDMA [7,116]. 5-APB has been shown to interact with the dopamine transporter, slowing dopamine reuptake and causing its reverse transport at high doses, and is an agonist at the 5-HT_2A_ and 5-HT_2B_-receptors in the rat. The interaction with serotoninergic receptors might mediate the hallucinogenic and cardiotoxic effects [152].

Stimulants of the thiophene designer drug groups have been shown to interact with 5-HT adrenergic and dopaminergic receptors, as well as N-methyl-D-aspartate (NMDA) and sigma-1 receptors [7]. The locomotor sensitization effect might be mainly mediated by dopaminergic activation, as shown for metathiopropamine (MPA), an NPS of the methamphetamine type, the effect of which is reversed by D_2_ but not by D_1_ receptor antagonists [156].

Psychedelics and hallucinogen determine alterations in the perception, beside mood and cognition modifications [157]. Within this class, tryptamines, e.g., N,N-dimethyltryptamine (DMT) and psilocybin, and “psychedelic amphetamines,” e.g., 2,5-dimethoxy-4-iodoamphetamine (DOI) and N-benzylphenethylamines (NBOMes), are included [7,158]. Neuropsychological effects of many psychedelics, including the head twitch response, which is used as a behavioral paradigm to distinguish hallucinogenic drugs, are mediated by the activation of 5-HT receptors, for which NBOMes show high affinity [158]. Generally, phenethylamines, also called “party pills” [158], such as 25B-NBOMe, have high 5-HT_2A_ and 5-HT_2C_ affinity and potency [158]. However, many NBOMEs also display affinity for dopaminergic receptors, e.g., D_2_, and for monoamine transporters, leading to abuse potential and rewarding and reinforcing effects [159,160,161]. Substituted phenethylamines, such as MAL and BOD, also alter the dopaminergic system by interacting with receptors in the nucleus accumbens and dopamine transport [162]. In addition, 4-iodo-2,5-dimethoxy-N-(2-methoxybenzyl)phenethylamine (25I-NBOMe) increases glutamate levels [7]. Although mainly mediating serotoninergic action, most tryptamine bind to 5-HT_1A_ receptors. Moreover, as demonstrated by in vitro studies, they bind on adrenergic, dopaminergic, and histaminergic receptors and transporters. For example, psilocin is a transporter inhibitor, while DMT is a transporter substrate [7].

Another class of NPS, properly of the dissociative type, is represented by derivative of phencyclidine (PCP) and ketamine, which are N-methyl D-aspartate (NMDA) receptor antagonists. Subjective effects associated with the intake of these drugs include dissociative-like effects, with alteration of the mood and thought, and schizophrenia-like effects [163].

Antidepressant effects of these compounds, e.g., methoxetamine, as demonstrated by forced swim tests on mice, might be related to the interactions with the glutamatergic system by the activation of the mammalian target of rapamycin, involved in synaptic plasticity, by a modulation of the brain-derived neurotrophic factor (BDNF), or by SERT properties. Moreover, methoxetamine has shown to be a DAT inhibitor and an agonist of muscarinic cholinergic and 5-HT2 receptors, and to produce analgesia [164]. Diphenidine and methoxphenidine are also dissociative drugs, acting as NMDA antagonists. Diphenidine further inhibits NET and DAT, while it is a less potent DAT inhibitor, but both do not mediate an efflux of monoamines [137]. N-Ethyl-1,2-diphenylethanamine (ephenidine) also acts selectively by blocking NMDA receptors with a higher potency than ketamine, though also interacting with NET and DAT, which might contribute to the behavioral profile of the drug [163].

## 4. Discussion and Conclusions

Harmful effects of NPS have been repeatedly proven to be fatal as reported by case reports, case series, and reviews present in the literature [7,85,165,166,167,168]. Also, data from studies applied to animals, conducted so far mostly in mice, unequivocally draw great attention to the acute toxic effects of these chemicals [169,170,171]. The thorough review on existing data carried out in this study confirms that the group of NPS is extremely large and variated. The review also shows that a distinction among different structural classes is fundamental to understand the pharmacological effects and to help clinicians in the diagnostic process in case of first aid admission.

Most importantly, our review demonstrates that the type of activity in the central nervous system as well as in periphery is not constant and homogeneous across different molecules [7]. Even within the same structural class, notwithstanding the identification of a primary or more typical mechanism of action, e.g., via the CB_1_ or CB_2_ receptors for synthetic cannabinoids [51], the effects might be mediated by different systems [103,104,105,106]. The unique profile of a single substance is responsible for its very peculiar toxicity, which might be strongly influenced by even minor structural or chemical modifications. This is particularly true not only for psychostimulants, psychedelics, and hallucinogens, which are composed by several subclasses [7,119,120,121,122,123], but also for synthetic opioids based on the differential elicitation of μ, κ, and δ opioid and nonopioid receptors [7,21,37]. This wide extent of possible neuromodulators and the great variability in action within a single class or subclass probably explains the higher toxicity of NPS when compared to the classical drugs of abuse and bares several consequences not only for clinicians, which could face unexpected effects and failure of classical treatments, but also for forensic toxicologists [20,86].

A deep study of each single compound, including its metabolism, which should be considered in the toxicological profile, as shown for several synthetic cannabinoids remaining active at the CB receptors, appears essential to understand its pharmacodynamic properties and true toxic potential. A similar comprehension can only be based, as shown in the present review, on the application of different types of studies, from preclinical studies, including in silico, in vitro, and animal studies, to human experiments and even to the application of innovative technologies, e.g., positron emission tomography (PET) and functional MRI [20]. A better knowledge of the pharmacokinetic and pharmacodynamic of NPS appears to be of fundamental importance to identify possible psychoactive metabolites, contributing to the toxicity and to pharmacological effects.

Finally, the forensic scientific community should devote more efforts toward developing and applying screening methods based on mass spectrometry detection which are able to identify the widest range of NPS in biological fluids in the setting of clinical and forensic toxicology, as well as to publishing more papers on this issue in medicolegal Journals [20,86,166,172,173]. In fact, one of the main issues is the apparently lack of toxicological data on users at first aid admission in the case of acute intoxication, with diagnosis based mainly on symptoms and reports [20]. Moreover, a comprehensive toxicological screening is not always applied in cases of overdoses due to the great analytical challenges posed by NPS identification and quantitation in biological fluids. All these issues contribute to an underestimation of the diffusion of the NPS among the population. Finally, an evaluation of the chronic effects of these chemicals is lacking, as well as the long-term effects deriving from mixing them.
